# Percutaneous endoscopic treatment for cervical ligamentum flavum gouty tophus: A case report

**DOI:** 10.1097/MD.0000000000015665

**Published:** 2019-05-17

**Authors:** Lin Xie, Xiang Zhang, Zhipeng Xi, Jingchi Li

**Affiliations:** a Department of Spine Surgery, Third Clinical Medical College of Nanjing University of Chinese Medicine; b Department of Orthopedic Surgery, Jiangsu Province Hospital on Integration of Chinese and Western Medicine, Nanjing, Jiangsu, China.

**Keywords:** cervical ligamentum flavum gouty tophus, minimally invasive, percutaneous endoscope

## Abstract

**Rationale:**

Cervical ligamentum flavum gout (CLFG) is relatively rare, and its clinical manifestations are complicated; hence, it is often confused with ligamentum flavum ossification. Gout tophi may relate to certain risk factors, such as renal insufficiency and lack of long-term effective uric acid treatment.

**Patient concerns:**

A 73-year-old man had a half-year history of left upper extremity pain and numbness, which was aggravated 6 months ago.

**Diagnoses:**

Computed tomography (CT) indicated spinal stenosis at the level of C5/6. Cervical stenosis was believed to be mostly related to the ossification of ligamentum flavum. The histological examination of the material removed during the surgery revealed fibrous tissues with pools of amorphous debris having a foreign body giant cell reaction, which is typical of urate gout.

**Interventions:**

We performed complete decompressions for this case with CLFG using posterior percutaneous endoscopic technique.

**Outcomes:**

The patient experienced a progressive improvement in the left upper extremity pain after the surgery, and no signs of cerebrospinal fluid leakage, infection, or other complications were experienced.

**Lessons:**

The clinician should include spinal gout in the differential diagnosis when dealing with patients with hyperuricemia, renal insufficiency, and axial pain with or without neurologic deficits. We have applied the percutaneous endoscopic technique for the treatment of spinal gout. It performed direct decompression with minimizing trauma and instability, which could be used as an alternative choice.

## 1 Introduction

Gout is monosodium urate, crystal-induced inflammatory arthritis associated with hyperuricemia. Renal insufficiency can develop in chronic hyperuricemia, and gouty tophus occurs commonly in locations where the blood circulation and temperature are quite low.^[1,2]^

Gout most commonly affects the peripheral joints in the upper and lower extremities. Less frequently, the condition could also involve the spine, with the possible incidence of 14% to 22%.^[3,4]^ Open surgery, usually in the form of laminectomy, seems to be the main course of treatment, particularly in patients with neurological deficits. Percutaneous endoscopic technique is routinely performed for disc herniation and spinal canal stenosis at our department.

We have presented here the case of a patient with cervical tophaceous gout involving the ligamentum flavum, who was treated with percutaneous endoscopy. To the best of our knowledge, this is the first case report of its kind.

## 2 Case report

A 73-year-old man had a half-year history of left upper extremity pain and numbness, which was aggravated 6 months ago. In a local hospital, magnetic resonance imaging (MRI) was performed to reveal cervical degeneration and hypertrophic ligamentum flavum at the level of C5/6. The discs of C3/4, C4/5, C5/6, and C6/7 exhibited posterior bulge. He had received conservative treatments, including physical therapy, oral nonsteroidal anti-inflammatory drugs, and steroids; however, the pain and numbness were not alleviated. Subsequently, the patient visited our hospital for further treatment. He denied any history of trauma, fall, fever, chills, night sweats, or gout. However, he had a 5-year history of hypertension and a 2-year history of type 2 diabetes mellitus.

On physical examination, the left spurling test was positive. In addition, the patient had decreased sensation in the left upper extremity. His Japanese Orthopedic Association (JOA) score was 13, and the visual analog score (VAS) of the neck and limb pain was 8.

Laboratory examination revealed leukocytosis of 9.33 × 10^9^/L (reference range 3.5 − 9.5 × 10^9^), urate level of 549 μmol/L (reference range 202–417), CRP of 0.88 mg/L (reference range 0–4.0), ESR of 4.0 mm/h (reference range 0–15.0), BUN 14.53 mmol/L (reference range 2.86–8.21), and creatinine level of 176 umol/L (reference range 59–104).

**Figure 1:**
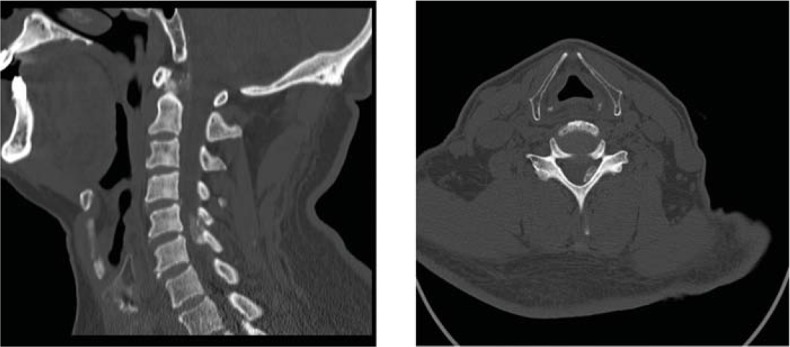
Preoperative images of CT showing spinal stenosis at the level of C5/6, mostly on left (due to the poor quality images obtained, the cervical stenosis was thought to be mostly ossification of ligamentum flavum, with no suspicion of intraspinal gout).

Computed tomography (CT) of the cervical spine indicated spinal stenosis at the level of C5/6, mostly on the left (Fig. 1). Owing to poor image quality, cervical stenosis was believed to be mostly related to the ossification of ligamentum flavum, with no suspicion of intraspinal gout.

**Figure 2:**
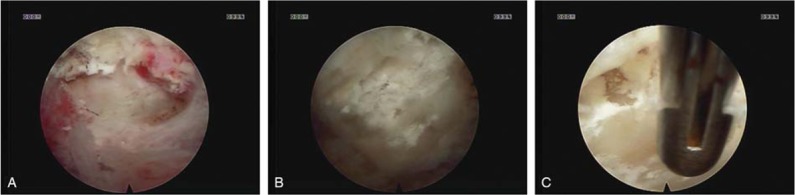
(A) An abnormal mass with a white, chalky, cheese-like and granular appearance was observed; (B) then ligamentum flavum was resected with an endo-punch; (C) the ligamentum flavum gouty was resected totally, and the dural sac was exposed.

Posterior percutaneous endoscopic surgery was performed in the prone position under general anesthesia with padding of all pressure points. Using fluoroscopic guidance, a left-sided longitudinal 7-mm skin incision was created above the facet joint at the level of C5/6. A dilator was bluntly inserted toward the lateral edge of the left C5/6 interlaminar space. After confirming the correct placement of the dilator by fluoroscopy, an operating sheath with a beveled opening was inserted through the dilator. Later, the dilator was removed, and the operating sleeve was inserted using the endoscope. The entire procedure was performed under direct visual control with neuromonitoring. The medial margin of the facet joint was removed by an endoscopic drill, and the ligamentum flavum was exposed. An abnormal mass with a white, chalky, cheese-like, and granular appearance was observed (Fig. 2A). The mass was removed with the aid of a grasper and bipolar radiofrequency. Then, the ligamentum flavum was resected with an endo-punch (Fig. 2B). Finally, the dural sac was exposed, and its pulsation was found to be good (Fig. 2C). The exiting nerve root felt free when palpated with a nerve hook. After removing all the instruments, the incised wound was sutured. The patient was advised to wear a neck collar for ≥weeks.

**Figure 3:**
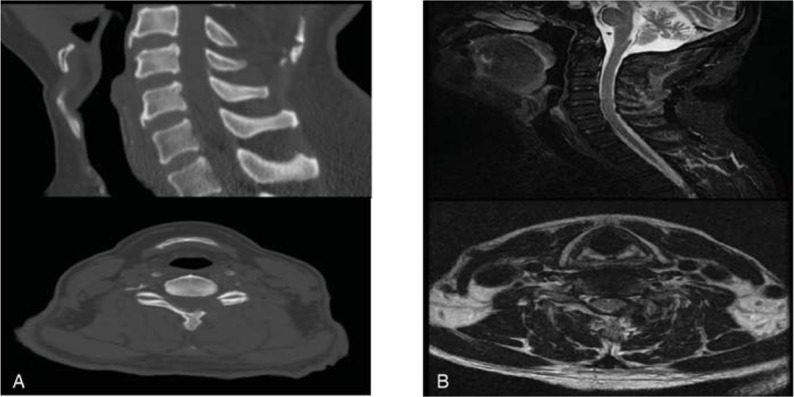
Postoperative images of CT (A) and MRI (B): postoperative images showed abnormal ligamentum flavum were removed, indicating a significant decompression.

**Figure 4:**
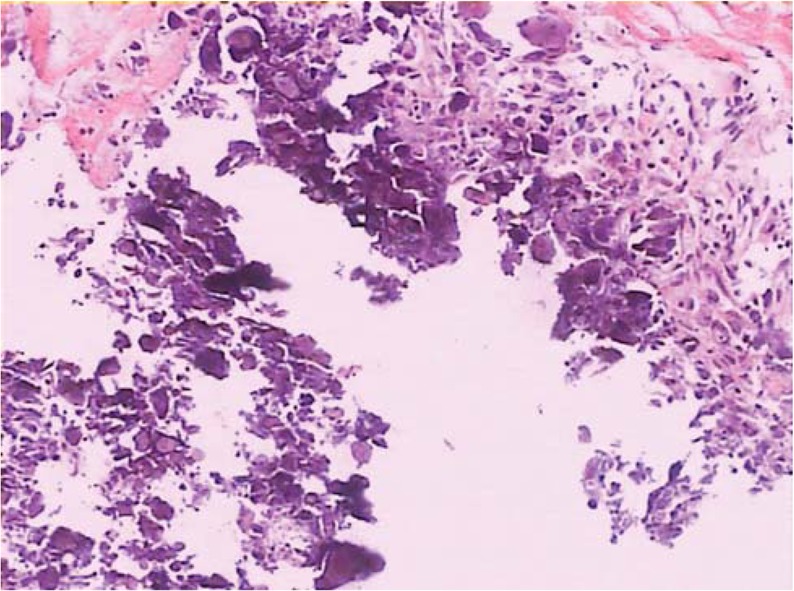
Histopathology section showing fibrous tissue with pools of amorphous debris with a foreign body giant cell reaction, which is typical of urate gout.

The patient experienced a progressive improvement in the left upper extremity pain after the surgery, and no signs of cerebrospinal fluid leakage, infection, or other complications were experienced. Postoperative images confirmed the removal of the abnormal ligamentum flavum, which resulted in a significant decompression (Fig. 3). A histological examination of the material removed during the laminectomy revealed fibrous tissues with pools of amorphous debris having a foreign body giant cell reaction, which is typical of urate gout (Fig. 4). The JOA score increased to 16, and the VAS decreased to 2 when the patient was discharged 3 days after the surgery. Clinical follow-ups after 3 and 6 months exposed no relapse of the symptoms.

This study was reviewed and approved by the Medical Ethics Committee of medical ethics committee of the Third Clinical Medical College of Nanjing University of Chinese Medicine. Written informed consent was obtained from the guardian of the patient for publication of the case report and any accompanying images.

## 3 Discussion

This report describes a relatively rare case of tophaceous gout that was deposited in the cervical ligamentum flavum. Initially, the patient was believed to probably have an ossification of ligamentum flavum or spinal stenosis, as inferred from the CT scan, and no peripheral tophi. Nevertheless, we eventually reached an appropriate diagnosis on the basis of pathological examination. Being familiar with the risk factors and radiological features are useful in making the correct diagnosis.

Tophaceous gout is often associated with heredity, environment, individual constitution, and metabolic disorders. The main risk factors include renal insufficiency, hypertension, chronic diuretic therapy, and the lack of long-term effective uric acid treatment.^[5]^ Renal insufficiency is commonly found among patients with gout.^[6]^ In our specific case too, the patient had a history of this condition. Laboratory test yielded BUN of 14.53 mmol/L (reference range 2.86–8.21) and creatinine of 176 umol/L (reference range 59–104), which indicated moderate renal insufficiency.

Inadequate kidney functioning may lead to an increase in the blood uric acid and creatinine level. Similarly, increased blood uric acid and creatinine may induce deterioration of renal functioning.^[6]^ Thus, lowering uric acid can result in a prompt and complete recovery of renal activity.^[7]^

The noninvasive diagnosis of spinal gout is mainly based on imaging techniques, including MRI, CT scan, and dual-energy computed tomography (DECT). The key imaging manifestations of spinal gout are erosions (41.5%), occupying lesions (23.9%), and degenerations (14.1%), which lack specificity, as demonstrated by a recent review.^[8]^

Another review compared these imaging tests.^[9]^ MRI findings are often nonspecific, with only 21% of the cases unequivocally interpreted as spinal gout. Calcifications within the bony erosions are more prominently visible on CT scan. Among the scans performed on patients with spinal gout, 96.6% were read as abnormal, with 18.6% of the scans reported as being highly suspicious for tophaceous gout. DECT scans could help with the diagnosis as they more effectively differentiate tophi from other types of masses. However, these scans may be less sensitive in identifying diffuse as opposed to dense lesions.

Ligamentum flavum gout could easily result in neurological deficits as it is located adjacent to the spinal cord and nerve root.^[10]^ Clinically, ligamentum flavum is often confused with ossification of ligamentum flavum. In this case, the results of the CT scan suggested ossification of ligamentum flavum at the level of C5/6. Hence, it is difficult to differentiate between these 2 diseases. DECT may be an optional method to discern the differences as it distinguishes urate from calcification.

The gold standard of the diagnosis is pathological examination. A definitive diagnosis is usually made during surgery, which typically shows chalky white nodules or masses visualized macroscopically and negatively birefringent urate crystals observed microscopically under polarizing conditions.^[9]^

Although a recent study implied that medication can be as effective as surgical treatment,^[9]^ the first-line therapy is surgery in 61% to 77%^[9,11]^ of the spinal gout cases reviewed in the literature. Surgery is most common in cases with pressing neurological symptoms, especially when there is an evidence of spinal compression or when the differential diagnosis is suspicious of occupying lesions or erosions, such as infections or tumors. Surgery routinely involves a laminectomy or decompression, which is sometimes associated with fusion. In our case, we adopted the posterior percutaneous endoscopic technique, and, to the best of our knowledge, our work is the first such report.

For the first time, Ruetten^[12]^ published a technique for cervical disc herniation using posterior percutaneous endoscopic cervical discectomy. As the method offers a clear visualization of normal and pathological tissues, it poses less trauma to the paraspinal muscle, has lower complication rates, ensures faster return to day-to-day activities, and has attracted public attention.^[13,14]^ With the advancements in technology and the accumulation of experience, this technique can be applied for the treatment of osseous foraminal stenosis^[15]^ and shwannoma.^[16]^

We employed the posterior percutaneous endoscopic technique to treat patients. Upon comparison with the traditional open surgery, this method greatly reduces the damage to posterior ligaments, muscles, and bones while retaining maximal biomechanical stability of the cervical vertebrae. In addition, the treatment has the advantages of lowered blood loss, minimal postoperative pain, shorter hospitalization, and reduced costs.

Some surgeons have treated spinal gout through a minimally invasive approach using tubular retractors.^[17]^ The posterior percutaneous endoscopic technique provides better visualization by zooming in through the imaging system and effective hemostasis by employing the ligation system and bipolar radiofrequency.

## 4 Conclusions

The clinical manifestations of cervical ligamentum flavum gout are complicated and nonspecific; moreover, the spinal gout is relatively rare. When dealing with patients exhibiting hyperuricemia, renal insufficiency, and axial pain without or with neurological deficits, the clinician should include spinal gout in the differential diagnosis. We successfully applied the percutaneous endoscopic technique for the treatment of spinal gout. We performed direct decompression with minimal trauma and instability, which could be relied upon as an alternative choice. Nevertheless, the possibility of recurrence and the need for further observation remain unclear.

## Acknowledgments

The authors thank the reviewers for their helpful comments on this article and the patient for his participation and her agreement to publication of the report.

## Author contributions

**Conceptualization:** Lin Xie.

**Data curation:** Xiang Zhang.

**Formal analysis:** Zhipeng Xi.

**Funding acquisition:** Lin Xie.

**Project administration:** Lin Xie.

**Software:** Jingchi Li.

**Validation:** Lin Xie, Zhipeng Xi.

**Writing – original draft:** Xiang Zhang.

**Writing – review and editing:** Lin Xie, Zhipeng Xi, Jingchi Li.

## References

[1] Keith MP Gilliland WR Updates in the management of gout. Am J Med 2007;120:221–4. 17349440 10.1016/j.amjmed.2006.02.044

[2] Pascual E Andrés M Vela P Criteria for gout diagnosis? J Rheumatol 2013;40:356–8. 23547255 10.3899/jrheum.130001

[3] Konatalapalli RM Demarco PJ Jelinek JS Gout in the axial skeleton. J Rheumatol 2009;36:609–13. 19208604 10.3899/jrheum.080374

[4] Jajić I Gout in the spine and sacro-iliac joints: radiological manifestations. Skeletal Radiol 1982;8:209–12. 7112148 10.1007/BF00355508

[5] Sun M Vazquez AI Reynolds RJ Untangling the complex relationships between incident gout risk, serum urate, and its comorbidities. Arthritis Res Ther 2018;20:90. 29720278 10.1186/s13075-018-1558-3 PMC5932762

[6] Roughley MJ Belcher J Mallen CD Gout and risk of chronic kidney disease and nephrolithiasis: meta-analysis of observational studies. Arthritis Res Ther 2015;17:90. 25889144 10.1186/s13075-015-0610-9 PMC4404569

[7] Liu P Chen Y Wang B Allopurinol treatment improves renal function in patients with type 2 diabetes and asymptomatic hyperuricemia: 3-year randomized parallel-controlled study. Clin Endocrinol 2015;83:475–82. 10.1111/cen.12673 25400252

[8] Zhang T Yang F Li J Gout of the axial joint-A patient level systemic review. Semin Arthritis Rheum 2019;48:649–57. 29804629 10.1016/j.semarthrit.2018.04.006

[9] Toprover M Krasnokutsky S Pillinger MH Gout in the spine: imaging, diagnosis, and outcomes. Curr Rheumatol Rep 2015;17:70. 26490179 10.1007/s11926-015-0547-7

[10] Zheng ZF Shi HL Xing Y Thoracic cord compression due to ligamentum flavum gouty tophus: a case report and literature review. Spinal Cord 2015;53:881–6. 26078231 10.1038/sc.2015.93 PMC5399141

[11] Hasegawa EM de Mello FM Goldensteinschainberg C Gout in the spine. Rev Bras Reumatol 2013;53:296–302. 24051913

[12] Ruetten S Komp M Merk H Full-endoscopic cervical posterior foraminotomy for the operation of lateral disc herniations using 5.9-mm endoscopes: a prospective, randomized, controlled study. Spine 2008;33:940–8. 18427313 10.1097/BRS.0b013e31816c8b67

[13] Yong A Percutaneous endoscopic cervical discectomy using working channel endoscopes. Expert Revi Med Devices 2016;13:601. 10.1080/17434440.2016.1180245 27086505

[14] Liao C Qiang R Lei C Modified posterior percutaneous endoscopic cervical discectomy for lateral cervical disc herniation: the vertical anchoring technique. Eur Spine J 2018;1–9. 10.1007/s00586-018-5527-y 29478117

[15] Ye ZY Kong WJ Xin ZJ Clinical observation of posterior percutaneous full-endoscopic cervical foraminotomy as a treatment for osseous foraminal stenosis. World Neurosurg 2017;106:945–52. 28739520 10.1016/j.wneu.2017.07.085

[16] Ying GY Yao Y Shen F Percutaneous endoscopic removal of cervical foraminal schwannoma via interlaminar approach: a case report. Oper Neurosurg 2018;14:1–5. 10.1093/ons/opx088 29253290

[17] Vergara P O’Donovan DG Minimally invasive excision of lumbar tophaceous gout: case report. Int J Spine Surg 2017;11:37. 29372141 10.14444/4037 PMC5779265

